# The 2022 CDC opioid prescription guideline update: Relevant recommendations and future considerations

**DOI:** 10.1016/j.jdin.2023.07.006

**Published:** 2023-07-21

**Authors:** Ameya Gangal, Benjamin Stoff, Travis Blalock

**Affiliations:** a Department of Dermatology, Emory University School of Medicine, Atlanta, Georgia; b Emory Center for Ethics, Atlanta, Georgia

**Keywords:** dermatologic surgery, drug monitoring, hidradenitis suppurativa, opioid, pain management, prescriber habits

In November 2022, the Centers for Disease Control and Prevention updated a 2016 practice guideline for opioid prescription.^1^ This update consists of 12 key recommendations and five guiding principles related to clinical scenarios excluding sickle cell disease, nonsurgical cancer-related pain, palliative care, and end-of-life care. Dermatologists prescribe opioids in acute scenarios, including postoperatively following Mohs micrographic surgery and reconstruction, and for subacute or chronic pain related to inflammatory conditions, such as hidradenitis suppurativa, psoriasis, chronic ulcers, blistering skin diseases, and erosive lichen planus.

The 2022 Centers for Disease Control and Prevention guideline reiterates that clinicians should prescribe opioids at the lowest effective dose for the expected duration of pain warranting opioid use. The guideline also endorses patient-centered approaches to the initiation or taper of opioids. Clinicians should discuss benefits and risks of opioids and consider patient preference. The 2022 guideline further suggests that clinicians “evaluate benefits and risks with patients within 1-4 weeks of starting opioid therapy for subacute or chronic pain or of dosage escalation.” This recommendation is particularly relevant to the chronic management of patients who begin opioids for hidradenitis suppurativa, erosive lichen planus, or chronic ulcerating conditions, or even in the emergency department, where opioids are frequently prescribed for cellulitis and abscess.^2^ The 2022 guideline also recommends that clinicians maximize the use of nonopioid therapies, such as non-steroidal anti-inflammatory medications and topical anesthetics. Injectable anesthetics, such as bupivacaine, may also be used to reduce postoperative pain and narcotic use following Mohs micrographic surgery.^3^

Postsurgical pain management comprises the majority of opioid prescriptions among dermatologists.^4^ A 2018 study of prescriber data in a 1-year period revealed that 15% of dermatologists, primarily in surgical practices, prescribed greater than 10 opioid claims using Medicare Part D.^4^ In 2019, an expert panel convened to consider scenarios in which opioid prescription would be warranted for dermatologic procedures.^5^ The group assessed 87 common dermatologic procedures, concluding that most do not routinely require greater than 15 opioids (equivalent to 5 mg of oxycodone). Only reconstruction of full-thickness upper lip defects—the Abbe flap—routinely required between 1 and 15 opioids.^5^ While this consensus is invaluable to clinicians, recommendations are specific to procedural dermatology and provide no guidance on pain related to chronic skin disease.

An added 2022 recommendation outlines the use of prescription drug monitoring or high-sensitivity screening questions to mitigate opioid prescriptions to patients at higher risk for substance use. This recommendation is accompanied with the caveat that prescription drug monitoring data should be used with “all patients rather than differentially on the basis of assumptions about what [clinicians] will learn about specific patients.” The 2022 guideline also uses five new guiding principles, which emphasize an individualized, flexible, and multidisciplinary approach to opioid prescription. The fifth guiding principle encourages culturally informed communication to promote access to affordable, diversified pain management regimens and recognizes the role of bias in pain management. The 2022 guideline update is an important step toward thoughtful, safe, and patient-centered opioid stewardship. Dermatologists should continue to build on this national policy update and consider more specific opioid appropriateness recommendations for chronic, nonsurgical dermatologic conditions.

## Conflicts of interest

None disclosed.
